# A systematic evaluation of Dutch large language models’ surprisal estimates in sentence, paragraph and book reading

**DOI:** 10.3758/s13428-025-02774-4

**Published:** 2025-08-18

**Authors:** Sam Boeve, Louisa Bogaerts

**Affiliations:** Department of Experimental Psychology, Ghent University, Ghent, Belgium

**Keywords:** Reading, Prediction, Eye movements, Large language models, Dutch

## Abstract

Studies using computational estimates of word predictability from neural language models have garnered strong evidence in favour of surprisal theory. Upon encountering a word, readers experience a processing difficulty that is a linear function of that word’s surprisal. Evidence for this effect has been established in the English language or using multilingual models to estimate surprisal across languages. At the same time, many language-specific models of unknown psychometric quality are made openly available. Here, we provide a systematic evaluation of the surprisal estimates of a collection of large language models, specifically designed for Dutch, examining how well they account for reading times in corpora of sentence, paragraph and book reading. We compare their performance to multilingual models and an N-gram model. While models’ predictive power for reading times varied considerably across corpora, GPT-2-based models demonstrated superior overall performance. We show that Dutch large language models exhibit the same inverse scaling trend observed for English, with the surprisal estimates of smaller models showing a better fit to reading times than those of the largest models. We also replicate the linear effect of surprisal on reading times for Dutch. Both effects, however, depended on the corpus used for evaluation. Overall, these results offer a psychometric leaderboard of Dutch large language models and challenge the notion of a one-size-fits-all language model for psycholinguistic research. The surprisal estimates derived from all neural language models across the three corpora, along with the code to extract the surprisal, are made publicly available (https://osf.io/wr4qf/).

**Supplementary Information:**

The online version contains supplementary material available at 10.3758/s13428-025-02774-4.

## Introduction

Readers are known to experience greater processing difficulty when the word they are fixating on is less predictable given the preceding words. Over the years, growing evidence has supported this notion of probabilistic inference during language processing (Staub, 2024). This conception of reading as a continual process of forming and testing hypotheses on the identity of the next word was dubbed ‘a psycholinguistic guessing game’ (Goodman, 1967). The questions of how and why readers engage in some form of probabilistic inference ignited a debate that has continued to this day (Federmeier, 2007; Huettig, 2015; Pickering & Gambi, 2018; Pickering & Garrod, 2007; Ryskin & Nieuwland, 2023). The most influential theory of probabilistic inference is *surprisal theory* (Hale, 2001; Levy, 2008). This theory postulates a logarithmic relationship between a language unit’s contextual probability and the cognitive effort necessary for its processing. It has been implemented as readers creating a ranked set of possible sentence continuations. The difficulty of processing a new word is then related to the amount of reallocation necessary in the updated preference ranking (Levy, 2008). In other words, the more probability mass has to be shifted upon integrating a new word with the previous context, the more difficult this word will be to process (Staub, 2024). Formally, the impact a word has on the probability distribution over the next words can be expressed as the negative log of that word’s conditional probability, its surprisal value (Levy, 2008). As such, surprisal is put forward as the third key predictor in addition to the well-established effects of word frequency (Brysbaert et al., 2018) and word length (Hauk & Pulvermüller, 2004).

### Indexing word predictability

Multiple methods have been developed to estimate a word’s proposed processing difficulty (i.e. the surprisal value; Smith & Levy, 2013) using its conditional probability. Originally, word probabilities were estimated with the help of human raters, using the cloze task (Taylor, 1953). This has remained common practice (Schuster et al., 2021; Tiffin-Richards & Schroeder, 2020). Later, computational alternatives made their appearance with N-gram models and neural network models (Armeni et al., 2017). While the cloze procedure is time-consuming and results in potentially inaccurate estimates, especially for highly unlikely words (Smith & Levy, 2011), early computational methods, like recurrent neural networks, could only incorporate the near sentence context for the word’s probability estimate (De Mulder et al., 2015). This changed in 2017, when the transformer model was introduced (Vaswani et al., 2017). The architecture of a transformer model departs from previous probabilistic language models in significant ways, the primary one being the attention mechanism. This attention mechanism allows the models to break completely with the recurrence of earlier models. It functions to model precisely which of the previous words were most informative for next-word prediction, without diluting the influence of more distant words in the input sequence. As a result, the model learns to accurately represent between-word dependencies and contextual relationships across a wide context window. The base version of GPT-2, for example, has a context window of 1,024 words, while more recent models like Gemini 1.5 Pro have access to up to one million words. Additionally, by using positional encoding, the constraint to process input sequentially is loosened, opening up the possibility of parallelizing model training and increasing scaling capability (Vaswani et al., 2017).

### The explanatory power of predictability estimates from large language models (LLMs)

Intuitively, a transformer model equipped with an attention mechanism seems at odds with human cognition, as it models between-word relationships across a context window stretching far beyond any human’s working memory span. Still, a transformer model’s surprisal estimates have been shown to capture human judgments and reading time data for sentence processing better than other computational estimates. Goldstein et al. (2022), for example, found that GPT-2’s predictions correlated strongly with cloze ratings by native English speakers (*r* = 0.79) and that in 50% of the cases, both human and model assigned the same word the highest probability. In comparison, a 3-gram model only came up with a matching prediction in approximately 30% of the words. Similarly, the surprisal values of GPT-2 correlated more strongly with cloze probability than N-grams and long short-term memory networks (LSTMs) (Hao et al., 2020) while also matching the cloze probability distribution more closely (Eisape et al., 2020).

When it comes to reading time data, the evidence presents a more nuanced picture, with the advantage of transformer models stemming from more specific aspects of predictive processing. First, simulating reading times in English with the OB1-reader model, the inclusion of computational predictability estimates from transformer models (i.e. LLaMA and GPT-2) yielded a better fit than cloze probabilities (Rego et al., 2024). Additionally, comparing transformer models to recurrent neural networks (RNNs), Merkx and Frank (2021) concluded that the former’s surprisal estimates explained self-paced reading times and neural activation patterns better (but see Michaelov et al., 2024). Interestingly, when splitting the self-paced reading data into longer and shorter sentences, the benefit of transformer models was shown to stem primarily from longer sentences (Merkx & Frank, 2021). In a similar vein, de Varda et al. (2023) emphasized the importance of the processing measure itself: while the variance of first fixation durations was best explained by cloze surprisal, the late eye-movement measures (i.e. gaze duration, right-bounded time and go-past time) were captured better by transformer models’ surprisal estimates (de Varda et al., 2023).

These results highlight that transformer models are particularly effective at capturing the behavioural markers of processing difficulty associated with longer sentences and late eye-tracking measures. However, even within a certain eye-tracking measure (early or late), there is considerable variability in how well each model’s surprisal estimates account for these effects. Most notable is the so-called *inverse scaling trend* (Oh & Schuler, 2023; Oh et al., 2022). This refers to the observation that the surprisal estimates of larger transformer models (typically indexed by a model’s number of trainable parameters) fare worse at predicting reading times. This observation is thought to result from systematic biases in these larger models'probability estimates, specifically an underestimation of how surprising nouns and adjectives are coupled with an overestimation of the predictability of function words (Oh & Schuler, 2023). Yet the inverse scaling trend also depends to some degree on the processing measure being considered. Within a model family (e.g. gpt-neo) the surprisal estimates of the relatively larger models can show an advantage in predicting later eye-tracking measurements such as total reading time (de Varda & Marelli, 2023). Additionally, the method of deriving next-word probabilities also influences the fit to reading times. Kuribayashi et al. (2025) found that surprisal estimates extracted from a larger LLM’s internal layers aligned with reading behaviour as well as or better than those from smaller LLMs. Finally, there is a notable disconnect in trends observed for behavioural data and neural data. When predicting brain activation patterns from internal model representations, larger models do show an advantage over their smaller counterparts (Antonello & Huth, 2022; Caucheteux & King, 2022; Schrimpf et al., 2021). When, on the other hand, using surprisal directly to model the brain’s hemodynamic response, the same inverse scaling trend as for reading times emerges (Lin & Schuler, 2025). Combined, it appears that surprisal estimates taken from transformer models are especially well positioned to capture the intermediate to late integrative processes related to word predictability (de Varda et al., 2023), with the predictability estimates of GPT-2 (small version, 124 million parameters) occupying the sweet spot in predicting reading behaviour (Oh & Schuler, 2023; Shain et al., 2024).

As GPT-2’s surprisal estimates show a good fit to reading times, several recent studies have leveraged it for testing specific hypotheses on predictive language processing and refining surprisal theory. We discuss here a few examples, all using English models and reading data from native English speakers. First, using GPT-2’s predictions, Cevoli et al. (2022) found an interaction between surprisal and entropy (i.e. expected surprisal, uncertainty over potential sentence continuations) during natural reading, with target words high in surprisal being skipped less often in high entropy contexts. This could mean that a word’s entropy is taken as a clue by readers to determine the amount of parafoveal processing to engage in (Cevoli et al., 2022). Heilbron et al. (2023) tested this proposed integration of prediction and parafoveal preview by constructing a Bayesian ideal observer using the predictions of GPT-2 as a prior. This integration increased the amount of information that was extracted from the preview. Surprisingly, such an enhanced preview provided a worse fit to human reading times than an independent preview and prediction effect (Heilbron et al., 2023). Similarly, Hahn et al. (2022) used the predictions of GPT-2 for the implementation of a resource-rational model of sentence processing. In this model, a prediction is formed over an imperfect memory representation of the sentence context, showing that it can account for the difficulty experienced when processing nested recursive structures better than classic surprisal theory (Hahn et al., 2022). Overall, neural language models have been thoroughly evaluated, and the accessibility of these models allows them to be easily applied to test hypotheses on predictive language processing. Nevertheless, all evidence presented here stems from English models (mainly GPT-2) and a handful of corpora with reading data from native English speakers, including Dundee (Kennedy et al., 2013), Provo (Luke & Christianson, 2018) and Natural Stories Corpus (Futrell et al., 2021), among others.

### Current study: Moving beyond English

Transformer models offer a valuable resource with which to study the effects of predictability on natural language comprehension, and with the help of ecosystems as *Hugging Face*,^1^ they have become highly accessible. Despite the proliferation of language models, their development remains predominantly focussed on the English language, while language-specific models for other languages are of unknown psychometric quality. This asymmetry presents a significant methodological constraint for conducting psycholinguistic research in low(er)-resource languages. Large cross-linguistic comparisons are forced to rely on multilingual models (de Varda & Marelli, 2022, 2023; Wilcox et al., 2023; Xu et al., 2023), although there are studies using language-specific models (Oh & Schuler, 2025). Multilingual models are trained simultaneously on multiple languages, capturing cross-linguistic regularities. These models, however, risk not representing all languages equally well due to asymmetries in the training data and interactions between learned regularities (i.e. catastrophic forgetting). For a fixed model size, increasing the number of languages during training increases the cross-linguistic performance up to a breaking point, after which both monolingual and multilingual performance declines, a trade-off referred to as the *curse of multilinguality* (Conneau et al., 2020). Multilingual models such as BERT-multi and XLM have demonstrated the ability to predict reading times across Dutch, German, English and Russian (Hollenstein et al., 2021). Additional work using a multilingual model to estimate surprisal showed a consistent effect of surprisal on reading times across languages, although the size of the effect varied considerably (de Varda & Marelli, 2022; Wilcox et al., 2023). This raises the question of whether this variability is language-specific or an artefact of the model used to quantify surprisal. This concern is backed by evidence showing that the linearity of the surprisal effect is unstable and depends on the language model used to estimate the surprisal. For Dutch, surprisal estimates of the multilingual model mGPT showed a sublinear trend, while a monolingual model showed a superlinear trend (Xu et al., 2023).

The complexity of choosing a suitable, language-specific model is exacerbated by the growing base of evidence that a model’s language ability, indexed by standard natural language processing (NLP) benchmarks (if these exist for the language under study) such as GLUE (General Language Understanding Evaluation; Wang et al., 2019), BLiMP (Bechmark of Linguistic Minimal Pairs; Suijkerbuijk et al., 2024; Warstadt et al., 2023) or MSGS (Mixed Signals Generalization Set; Warstadt et al., 2020), does not reflect the degree to which the model can account for psycholinguistic occurrences such as reading times (Steuer et al., 2023).

In this work, we aim to close this gap for Dutch. We present a comprehensive evaluation of 14 Dutch transformer models, five multilingual transformer models and an N-gram model, examining these models’ ability to explain variability in eye movements when reading individual sentences, paragraphs and a full novel. Our analysis draws upon three distinct eye-tracking corpora (Radboud Coregistration Corpus of Narrative Sentences [RaCCooNS], Multilingual Eye-movement Corpus [MECO] and Ghent Eye-Tracking Corpus [GECO], respectively; see Method section), each featuring different types of text content and varying amounts of context. Furthermore, we test whether established phenomena from English language modelling, specifically the inverse scaling trend and the linear effect of surprisal on reading times, generalize when assessed using Dutch LLMs.

Through this systematic evaluation, we provide researchers with empirically grounded guidance for model selection in Dutch psycholinguistic studies. This facilitates investigations of word predictability and surprisal theory beyond English-centric frameworks and sheds light on how multilingual models compare to language-specific models for psycholinguistic purposes. In this spirit, we make the surprisal estimates for each corpus’ word-by-word reading data and the method used to extract the surprisal values publicly available (https://osf.io/wr4qf/). As the first large-scale psychometric LLM comparison for Dutch, we show the value of these models in three different corpora. Additionally, by listing the large amount of language-specific Dutch LLMs available online, we highlight that surprisal can be investigated in many languages without having to resort to potentially suboptimal multilingual models, facilitating a more general understanding of surprisal theory.

## Method

### Language models

We selected language models using the Dutch large language modelling leaderboard (Vanroy, 2023),^2^ which ranks models using existing NLP benchmarks (e.g. Measuring Massive Multitask Language Understanding [MMLU]; Hendrycks et al., 2021). The models retained for evaluation were limited to autoregressive models, a class of models that only makes use of the left context in predicting the next word, as opposed to masked language models (e.g. BERT), which have access to both left and right sentence context, making their surprisal estimates less psychologically plausible (Wilcox et al., 2023). We considered only models that were pre-trained on Dutch data or that were specifically adapted for the Dutch language. Notably, gpt2-small-dutch-embeddings, gpt2-medium-dutch-embeddings and gpt2-small-dutch are recycled from an English GPT-2 model (de Vries & Nissim, 2020). For the gpt2-small-dutch-embeddings and gpt2-medium-dutch-embeddings models, the lexical embedding layer was retrained for Dutch while keeping the transformer layers fixed, whereas for gpt2-small-dutch, the transformer layers were also fine-tuned on Dutch text (de Vries & Nissim, 2020). Similarly, tweety-7b-dutch-v24a, was developed using a specific cross-lingual transfer strategy whereby token embeddings of the new language are initialized as a weighted average of semantically similar tokens in the source language (Remy et al., 2024). See Table 1 for an overview of all models used in this study.

**Table 1:** Summary of all the models included in the study

Model name	Parameter count	Context length	Training type	Model family	Tokenizer	Reference/Link
*EuroLLM-9B*	9.2 billion	4,096	Pre-trained	Llama	LlamaTokenizer	Martins et al. (2024)
Llama-3-8B-dutch	8 billion	8,192	Fine-tuned	Llama	LlamaTokenizer	https://huggingface.co/ReBatch/Llama-3-8B-dutch
*salamandra-7b*	7.8 billion	8,192	Pre-trained	Llama	LlamaTokenizer	Gonzalez-Agirre et al. (2025)
tweety-7b-dutch-v24a	7.4 billion	8,192	Modified	Mistral	GPT2Tokenizer	Remy et al. (2024)
Boreas-7B	7.2 billion	2,048	Pre-trained	Mistral	LlamaTokenizer	https://huggingface.co/yhavinga/Boreas-7B
GEITje-7B	7.2 billion	8,192	Pre-trained	Mistral	LlamaTokenizer	Rijgersberg & Lucassen (2023)
fietje-2	2.8 billion	2,048	Pre-trained	Phi	CodeGenTokenizer	Vanroy (2024)
*salamandra-2b*	2.3 billion	8,192	Pre-trained	Llama	LlamaTokenizer	Gonzalez-Agirre et al. (2025)
*EuroLLM-1.7B*	1.7 billion	4,096	Pre-trained	Llama	LlamaTokenizer	Martins et al. (2024)
*mGPT*	1.4 billion	2,048	Pre-trained	GPT	GPT2Tokenizer	Shliazhko et al. (2023)
gpt-neo-1.3B-dutch	1.3 billion	2,048	Pre-trained	GPT-neo	GPT2Tokenizer	https://huggingface.co/yhavinga/gpt-neo-1.3B-dutch
gpt2-large-dutch	774 million	1,024	Pre-trained	GPT	GPT2Tokenizer	https://huggingface.co/yhavinga/gpt2-large-dutch
gpt2-medium-dutch	355 million	1,024	Pre-trained	GPT	GPT2Tokenizer	https://huggingface.co/yhavinga/gpt2-medium-dutch
gpt2-medium-dutch-finetune-oscar	355 million	1,024	Fine-tuned	GPT	GPT2Tokenizer	https://huggingface.co/ml6team/gpt2-medium-dutch-finetune-oscar
gpt2-medium-dutch-embeddings	344 million	1,024	Modified	GPT	GPT2Tokenizer	de Vries & Nissim (2020)
gpt-neo-125-M-dutch	125 million	2,048	Pre-trained	GPT-neo	GPT2Tokenizer	https://huggingface.co/yhavinga/gpt-neo-125M-dutch
gpt2-small-dutch-finetune-oscar	124 million	1,024	Fine-tuned	GPT	GPT2Tokenizer	https://huggingface.co/ml6team/gpt2-small-dutch-finetune-oscar
gpt2-small-dutch	117 million	1,024	Modified	GPT	GPT2Tokenizer	de Vries & Nissim (2020)
gpt2-small-dutch-embeddings	117 million	1,024	Modified	GPT	GPT2Tokenizer	de Vries & Nissim (2020)
5-gram	–	4	Pre-trained	–	–	Frank & Aumeistere (2023)

Overview of Dutch language models meeting selection criteria, arranged in descending order by parameter count. The model names of multilingual models are shown in italics. The training type ‘modified’ refers to models that were recycled from an English model by retraining the lexical embedding layer (i.e. gpt2-small-dutch-embeddings, gpt2-medium-dutch-embeddings) with optionally a small amount of Dutch-specific fine-tuning (i.e. gpt2-small-dutch), or by using a specific trans-tokenization strategy enabling cross-lingual vocabulary transfer (i.e. tweety-7b-dutch-v24a). Context length is expressed in number of tokens. Details of the model configurations can be found using the model cards on the Hugging Face website

We restricted the search to models composed of less than 10 billion parameters to keep inference using these models as accessible as possible. Beyond the leaderboard, we searched the Hugging Face model repository for complementary models that adhered to our criteria (e.g. gpt-neo-125-M-dutch). As a comparison, we included five multilingual language models, namely mGPT (Shliazhko et al., 2023; used in Wilcox et al., 2023), two variants of a salamandra model (Gonzalez-Agirre et al., 2025) and two variants of EuroLLM (Martins et al., 2024). Finally, we included a 5-gram model (Frank & Aumeistere, 2023) as a comparison to a traditional language model based on co-occurrence counts. Note that not only does the number of trainable parameters vary significantly, but the context length, training procedure (including differences in the size of training data) and tokenizer differ significantly as well. A model’s context length denotes how many previous tokens can be considered for the prediction of the next token.

The tokenizer controls how words are converted to numerical data. At one extreme, a tokenizer might split text solely at word boundaries, while at the other extreme, the text can be split into individual characters. Most common are so-called subword tokenizers. The principle is that frequent words are represented as is, but infrequent words are split into smaller subwords (e.g. ‘tokenization’ split into ‘token’ and ‘ization’). The GPT2Tokenizer starts from individual characters and learns to merge those to represent all words in the vocabulary with the least number of tokens. GPT2Tokenizer uses byte-level byte-pair encoding (BPE), allowing robust handling of diverse text and special characters but sometimes causing misalignment between tokens and natural word boundaries. The LlamaTokenizer also uses BPE but with a SentencePiece tokenizer. Unlike GPT-2’s deterministic BPE, it assigns probabilities to subwords and selects the most likely segmentation. A benefit of this approach is its flexibility, although it may result in less efficient token usage. Finally, CodeGenTokenizer uses an adapted BPE tokenization optimized to frequent patterns and structures in computer code (e.g. symbols and whitespace). More details on the (mis)alignment of tokens and word boundaries for each model’s tokenizer and each corpus can be found in the supplementary materials (Table S1). Throughout this paper, we use the term ‘words’ instead of ‘tokens’, as words serve as the meaningful units of evaluation in this study. It is important to note however that some words were processed by the models as multiple tokens. In these cases, the surprisal estimates of those tokens were summed to obtain a surprisal estimate for the entire word.

### Reading data

Three eye-tracking corpora consisting of or containing Dutch material were used to evaluate the psychometric quality of the Dutch LLMs. The corpora differed in the amount of text presented to readers, focussing on sentence reading, paragraph reading and the reading of a full book. Three different eye-movement patterns were considered for each corpus: first fixation duration, gaze duration and total reading time. A description of each measure is provided in Table 2.

**Table 2:** Description of the eye tracking measures

Measure	Description
First fixation duration (FFD)	The duration of the fixation landing on the target word during its first pass (excluding words that were skipped in the first pass)
Gaze duration (GD)	The total time spent fixating on a word during first-pass reading, thus before the gaze leaves it for the first time
Total reading time (TRT)	Summation of all fixation durations in the current word. This includes re-fixations on the target word after regressive saccades

#### RaCCooNS

The Radboud Coregistration Corpus of Narrative Sentences (RaCCooNS) is a corpus of simultaneously recorded eye tracking and electroencephalography (EEG) while participants read separate narrative sentences (Frank & Aumeistere, 2023). The corpus presents the data from 37 participants (mean age = 26.2 years) reading 200 sentences from the SONAR-500 corpus (Oostdijk et al., 2013). Sentences in this corpus were at least five words long and consisted of maximally 30 tokens. In total, the corpus presents word-by-word reading data for over 2,783 words. For the current study, we only used the eye-tracking data.

#### MECO

The Multilingual Eye-movement Corpus (MECO) offers reading data across 13 languages, including Dutch (Siegelman et al., 2022). The participants (*N* = 45, mean age = 22.69 years) read 12 Wikipedia-like expository paragraphs. The Dutch paragraphs were between 7 and 12 sentences long and contained minimally 161 and maximally 213 words. Combined, the Dutch part of the corpus consists of 2,231 words.

#### GECO

The Ghent Eye-Tracking Corpus (GECO) is a mono- and bilingual reading corpus reporting the eye-tracking data from participants reading a complete novel in Dutch and English (Cop et al., 2017). Participants (*N* = 19, mean age = 21.2 years) read the first half of the novel, *The Mysterious Affair at Styles* by Agatha Christie, in one language and the second half in the other language, the order of which was counterbalanced. We only use the data for participants reading in their first language (L1; Dutch). Combined, 10 participants read the first half of the novel in Dutch and a different group of eight participants read the second half in Dutch after reading the first half in English. In total, the corpus presents reading data for 59,716 words, with an average sentence length of 12 words.

### Surprisal estimates

Using the language models, the probability of each word conditioned on the preceding words was extracted with the Python package transformers (version 4.42.4). The probability estimation for each word was based on the maximal amount of context that could be accounted for by each model (i.e. the size of the context window, see Table 1). For the RaCCooNS and MECO data, this means that the probability estimate of a word was conditioned on all the previous words in a sentence or paragraph, respectively, as they never exceeded the maximal context size of the transformer models. For the GECO corpus, we opted for a sliding context window approach. First, we extracted the word probabilities based on the preceding words for as long as they fit in the context window. On the first token that exceeded the maximal context length, the context window was shifted in steps of half the maximal context window. As a result, all remaining word probabilities are conditioned on a textual input that is at least half the model’s total context window.

Next, considering the strong evidence for a logarithmic linking function between predictability and reading times (Smith & Levy, 2013; Wilcox et al., 2023), the probability estimates were transformed into surprisal values by taking the negative natural logarithm. If a model’s tokenizer split a word into multiple subword tokens, the log probabilities of these tokens were summed to obtain an estimate for the complete word.

### Analysis

#### Psychometric predictive power

First, we filtered out all words attached to a punctuation mark, including apostrophes and hyphens. We also excluded sentence initial words (Frank & Aumeistere, 2023) and words outside the 5-gram’s vocabulary, as for these words no probability estimate was obtained. Words with no fixations landing on it during first-pass reading (i.e. skips) were excluded from the analyses. This left us with 95,814, 67,595 and 549,220 data points for the RaCCooNS, MECO and GECO corpora, respectively.

The predictive power of the models'surprisal estimates for by-word processing difficulty was then evaluated for every corpus and for each eye movement metric (i.e. FFD, GD and TRT). To this end, we fitted a linear regression model for each combination of the eye-movement measures as dependent variables and a computational surprisal estimate as independent variable. Each regression model additionally included the following control measures: word frequency using the Zipf value taken from SUBTLEX-NL (Brysbaert et al., 2018; Keuleers et al., 2010), word length (in characters) and the position of the word in the sentence. As eye movements during reading are also sensitive to the characteristics of the previous word, known as spillover effects (Frank et al., 2013), each model included the word length, frequency and surprisal of the previous word.

In line with previous studies, the amount of additional variance in readers’ eye movement patterns explained by the inclusion of the surprisal estimates of each language model was quantified by comparing the full regression model to a baseline model that contained all dependent variables except the surprisal estimates.^3^ The predictive power of the surprisal estimates was tested using a tenfold cross-validation of the regression models. Regression coefficients of the predictors were estimated on nine folds of the data, while the quality of the fit was measured on the left-out fold (in line with previous studies; de Varda et al., 2023). On each of these folds the increase in log likelihood associated with including the surprisal estimates was assessed. To facilitate the comparison across the three corpora, the difference in log likelihood is expressed relative to the number of data points per corpus, thereby denoting the increase in log likelihood per word by including the surprisal estimate of the current and previous word. We report the average difference in log likelihood per word across the 10 folds. The *p* values for each fold were calculated using the likelihood ratio test and aggregated over folds using Fisher’s combined probability test. All analyses were conducted in R (version 4.4.1; R Core Team, 2024).

In our description of the results, we will focus on GD, as this is considered a cleaner measure of first-pass processing difficulty since it does not include regressive saccades such as TRT. The effects on FFD, on the other hand, are generally too early to show an influence of context and are mainly associated with orthographic processes and early lexical access (Radach & Kennedy, 2013).

#### Linking function surprisal and reading times

Previous studies have concluded that the linear linking function of surprisal and reading times generalized across languages (Wilcox et al., 2023). To test whether this linear relation holds when using specialized language models for Dutch, we use generalized additive models (GAMs), which are capable of modelling non-linear relationships. In this analysis we compare the link of surprisal and reading times for the surprisal of a multilingual model used in previous studies (mGPT; Wilcox et al., 2023), the best-performing multilingual model and the best-performing Dutch model across all three corpora in the previous analysis. This analysis will focus on gaze duration as dependent measure since this metric is thought to be most strongly associated with first-pass processing difficulty (Wilcox et al., 2020, 2023). First, all non-zero gaze durations on each word were averaged across participants to mitigate high by-participant variance potentially affecting the non-linear model fit. Next, in line with the previous analysis, sentence initial words and words attached to punctuation were omitted from the analysis. The resulting numbers of data points for each corpus were as follows: RaCCooNS, 2,315; MECO, 1,890; GECO, 45,390.

Testing the linearity of the surprisal effect entails comparing the difference in log likelihood of a linear and non-linear GAM relative to a shared baseline model. The non-linear GAM includes smooth terms for surprisal of the current and previous word while also including tensor product terms for the interaction between frequency (in Zipf) and word length of the current and previous word. The linear GAM includes a tensor product term for the interaction between frequency and word length but instead enforces a linear effect of surprisal (similar to regular linear regression). The linear and the non-linear models are then compared to a shared baseline model that only holds a tensor product term for frequency and word length.^4^ If the effect of surprisal on gaze duration is indeed linear, the non-linear GAM should not yield an advantage in log likelihood. Using a tenfold cross-validation, the log-likelihood differences relative to the baseline model were computed. With a paired permutation procedure (*N* = 1,000), we tested whether the mean difference in log-likelihood improvements between the linear and non-linear models (relative to the baseline) was significantly different from what would be expected under the null hypothesis that the two sets of improvements would be exchangeable. To visualize the relationship between surprisal and slowdown on gaze duration, we fit a linear and non-linear model on the 10 folds of the data and then sampled the models’ predicted reading times based on surprisal values between 0 and 20 (following Wilcox et al., 2023). GAMs were fitted using the mgcv package in R (version 1.9–1; Wood, 2017).

## Results

### Psychometric predictive power

**Fig. 1:**
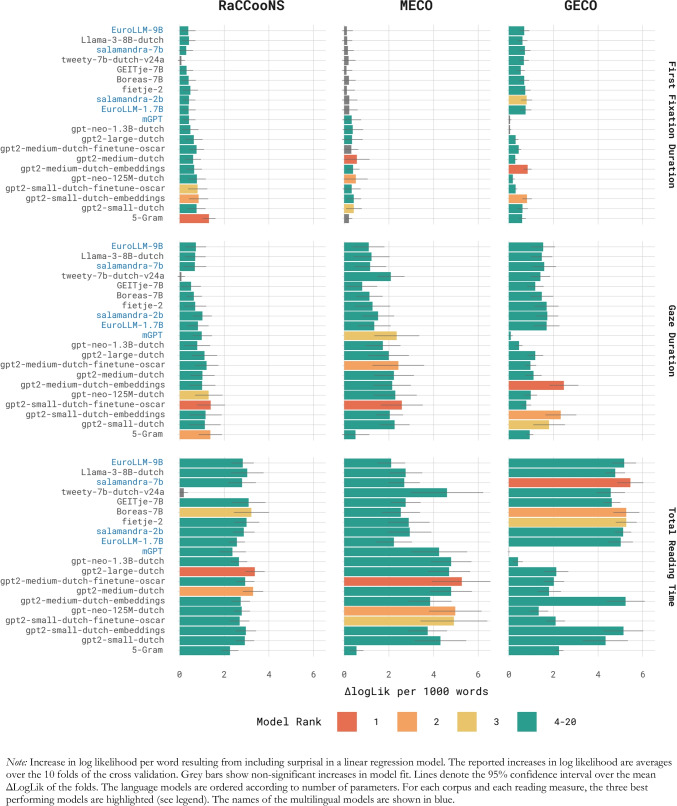
Models’ ability to predict reading times: Results of the cross validation

The results of the tenfold cross-validation are presented in Fig. 1. As expected, surprisal caused the largest increases in model fit for GD and TRT, while for FFD a substantial number of models’ surprisal estimates did not yield a significant benefit in model fit at the α = 0.05 level.

For each corpus and for each dependent variable, the best-performing model was an LLM specifically trained for Dutch, with the sole exceptions being the first fixation durations in the RaCCooNS corpus, where the N-gram’s surprisal estimates predicted reading times best, and total reading times in the GECO corpus, for which salamandra-7b performed best.

**Fig. 2:**
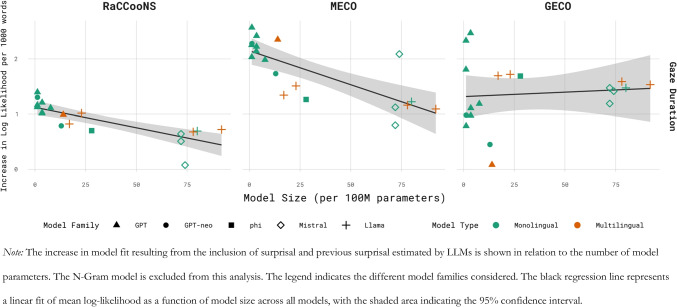
Psychometric predictive power in relation to model size for predicting gaze duration

Focussing on the results for GD, there are indications of an inverse scaling trend (better performance for smaller models) among the models considered here (see Fig. 2). This is especially the case for the RaCCooNS and MECO corpora, while for the GECO corpus this trend does not show as strongly. For the two smaller corpora, one of the smaller models’ surprisal estimates caused the largest increase in model fit. For the RaCCooNS and MECO corpus, gpt2-small-dutch-finetune-oscar (124 million parameters, context length of 1,024 tokens) was the best-performing model. For GECO, on the other hand, the multilingual salamandra-7b model (7.8 billion parameters, context length of 8,192 tokens) had the greatest psychometric predictive power. Overall, based on the sum of the model’s ranking, gpt2-small-dutch (117 million parameters, context length of 1,024 tokens), the smallest transformer model in this evaluation (parameter-wise), is the best choice for quantifying surprisal in Dutch text (ranked sixth for RaCCooNS, fifth for MECO and third for GECO). When considering the total increase in log likelihood per word across all three corpora, gpt2-medium-dutch-embeddings is the best choice (Σ(ΔLogLik per 1,000 words) = 5.62), although this mostly stems from its performance on the GECO corpus (ΔLogLik per 1,000 words = 2.47, *SE* = 0.28, *p* < 0.001).

Comparing models with the same type of architecture and a comparable training procedure confirms the inverse scaling trend for Dutch models, albeit with some exceptions. First, in the case of gpt-neo-125-M-dutch and gpt-neo-1.3B-dutch, two models differing only in the number of parameters, the smaller variant has greater predictive power for all three corpora. Next, for gpt2-medium-dutch and gpt2-large-dutch, two models that are both trained on the cleaned Dutch mc4 dataset (Raffel et al., 2023), the smaller model only shows a benefit on the MECO data, although both models are very similar overall in performance. Two additional *matched* models are gpt2-small-dutch-embeddings and gpt2-medium-dutch-embeddings, both modified versions of a gpt2-small and gpt2 base model (respectively, Radford et al., 2019). The smaller model only has an advantage on the RaCCooNS data, while the medium model’s surprisal estimates show a superior fit for the MECO and GECO data. A similar pattern is observed for the models gpt2-small-dutch-finetune-oscar and gpt2-medium-dutch-finetune-oscar, for which the small variant is superior on the RaCCooNS and MECO corpora but not on the GECO corpus. Figure 2 presents model performance as a function of the number of model parameters for each model family included in this study.

Overall, the five multilingual models tested in this comparison performed worse than their counterparts specifically trained on Dutch material. On the RaCCooNS data, the models mGPT, EuroLLM-1.7B, salamandra-2b, salamandra-7b and EuroLLM-9B clustered on the lower end of the performance distribution. The situation for MECO was similar, with the exception of a high third rank for mGPT. For GECO, mGPT’s surprisal estimates did not predict reading times, while the salamandra-2b and EuroLLM-1.7B models performed well (ranked fourth and fifth, respectively).

Considering the full collection of language models, it is notable that for the RaCCooNS and MECO data, the largest models (i.e. GEITje-7B, Boreas-7B, tweety-7b-dutch-v24a, Llama-3-8B-dutch, salamandra-7b and EuroLLM-9B) are clustered at the lower end of the ranking, while their surprisal estimates fit the GECO data noticeably better. A linear regression model was fit to examine the relationship between model size (in number of parameters) and the increase in fit to eye-tracking data with the inclusion of its surprisal. The linear model included main effects for corpus and eye-tracking measure (ET), as well as interactions between model size and both corpus and model family.^5^ This model showed a significant negative main effect of model size on log-likelihood improvement, indicating an inverse scaling trend. Specifically, each additional 100 million parameters was associated with a 0.023 decrease in log-likelihood gain per word in the corpus (*β* =  − 0.023, *SE* = 0.0078, *t*(159) =  − 2.94, *p* = 0.0038).

An overview of all estimates of predictive power for gaze duration with their respective confidence intervals can be found in the supplementary material (Table S2).

### Linking function surprisal and reading times

**Fig. 3:**
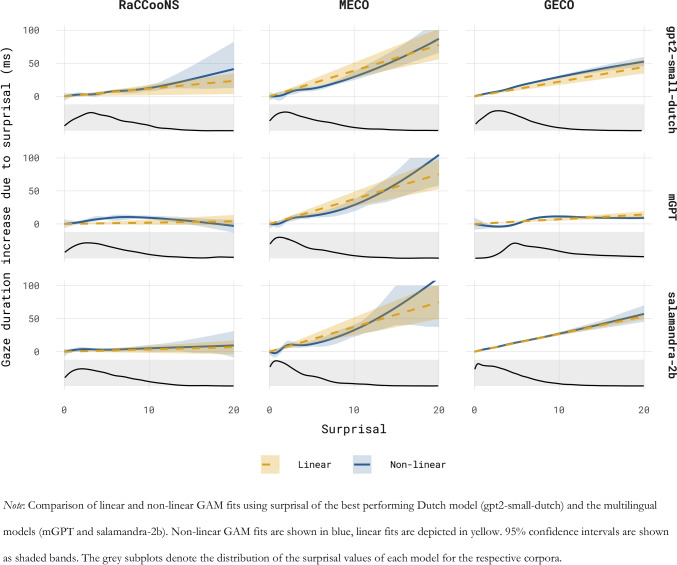
Linking function of surprisal and gaze duration

**Fig. 4:**
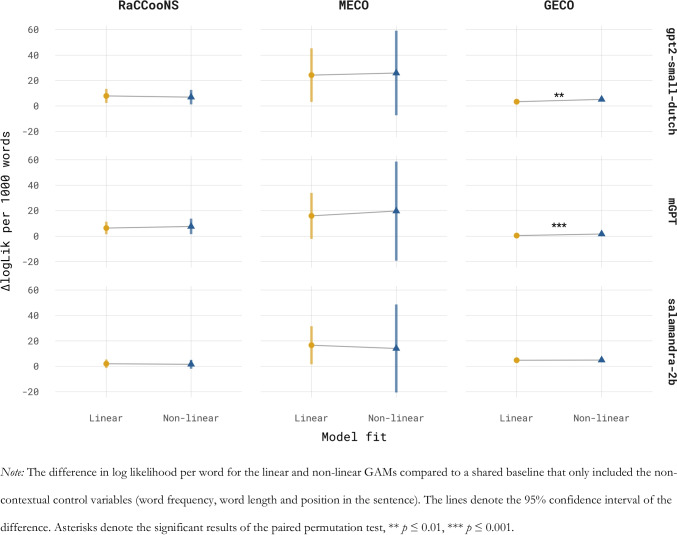
Comparison of linear and non-linear models

As gpt2-small-dutch was the best-ranked model for GD over the three corpora, we compare the link between surprisal and GD when surprisal is estimated using gpt2-small-dutch and when it is quantified using the multilingual model mGPT. Furthermore, as salamandra-2b was the best-performing multilingual model across all three corpora, we additionally analyse the linking function using salamandra-2b’s surprisal estimates. Figure 3 shows the GAM fits for the models allowing for a non-linear linking function and those enforcing a linear link. Overall, for each corpus and all three language models, reading times increase with increasing surprisal (see Fig. 3). For all models and on all three corpora, the inclusion of surprisal and previous surprisal (either linear or non-linear) significantly increased model fit to the average GD over a baseline model omitting surprisal. Visually, the non-linear GAM fits align reasonably well with their linear counterparts. For RaCCooNS and MECO, the paired permutation test indicated no evidence of an advantage for the non-linear model (RaCCooNS; gpt2-small-dutch: ΔLogLik linear = 0.0079, *SE* = 0.0028, ΔLogLik non-linear = 0.0070, *SE* = 0.0029, *p* = 0.83; mGPT: ΔLogLik linear = 0.0064, *SE* = 0.0025, ΔLogLik non-linear = 0.0077, *SE* = 0.0031, *p* = 0.34; salamandra-2b: ΔLogLik linear = 0.0020, *SE* = 0.0017, ΔLogLik non-linear = 0.0016, *SE* = 0.0018, *p* = 0.61; MECO, gpt2-small-dutch: ΔLogLik linear = 0.024, *SE* = 0.011, ΔLogLik non-linear = 0.026, *SE* = 0.017, *p* = 0.99, mGPT: ΔLogLik linear = 0.016, *SE* = 0.0092, ΔLogLik non-linear = 0.020, *SE* = 0.020, *p* = 0.28; salamandra-2b: ΔLogLik linear = 0.0017, *SE* = 0.0077, ΔLogLik non-linear = 0.0014, *SE* = 0.0018, *p* = 0.64). Surprisingly, on the GECO data and for both gpt2-small-dutch and mGPT, the permutation test showed that the observed differences in log likelihood relative to the shared baseline were different for the linear and non-linear models (GECO, gpt2-small-dutch: ΔLogLik linear: 0.0034, *SE* = 0.00082, ΔLogLik non-linear: 0.0052, *SE* = 0.00057, *p* = 0.003; mGPT: ΔLogLik linear: 0.00052, *SE* = 0.00017, ΔLogLik non-linear: 0.0017, *SE* = 0.00032, *p* < 0.001). In both cases, the non-linear model showed a significantly better fit to the reading times. These differences are visible in Fig. 3, with the non-linear fit of the gpt2-small-dutch surprisal estimates showing a superlinear relation to reading times and that of mGPT showing a sublinear relation at both ends of the surprisal distribution. For salamandra-2b on the other hand, the non-linear model did not show an advantage (ΔLogLik linear: 0.0048, *SE* = 0.00049, ΔLogLik non-linear: 0.0049, *SE* = 0.00051, *p* = 0.15). The differences in log likelihood of the linear and non-linear models compared to the baseline model with only non-contextual predictors (i.e. word frequency and word length) are shown in Fig. 4.

## Discussion

The large majority of evidence for the impact of word predictability on reading has been established in the English language, with recent work pointing to transformer models as a superior method for extracting surprisal estimates (e.g. de Varda et al., 2023; Eisape et al., 2020; Hao et al., 2020; Merkx & Frank, 2021). The broad class of LLMs has seen a rapid proliferation in the number of openly available models. For languages other than English, researchers have turned to multilingual models that are trained to capture the language statistics of multiple languages at once (Wilcox et al., 2023). Meanwhile, language-specific transformer-based language models beyond English have been developed, but the validity of these models’ surprisal estimates in accounting for reading behaviour remained unevaluated. In the current work we provided a comprehensive evaluation of the predictive power of multiple Dutch transformer models, compared to commonly used multilingual transformer models and an N-gram model, in explaining variability in eye movements during the reading of narrative sentences (RaCCooNS corpus), paragraphs from Wikipedia articles (MECO) and a full novel (GECO). Furthermore, we aimed to test whether established phenomena from English language modelling, the inverse scaling trend and the linear effect of surprisal on reading times, generalized to Dutch LLMs.

### The impact of word predictability or reading

At the conceptual level, our results underline once more the effect of predictability on reading times. For nearly all models and across the three corpora the index of word predictability given the preceding context significantly predicted reading behaviour after controlling for common control variables, frequency and word length. For the first time, this study demonstrates the effect for Dutch using various computational estimates. It is also the first systematic comparison of this effect across corpora of increasing context sizes, including the reading of an entire book.

### Which Dutch model should one choose?

The answer here is clearly ‘it depends’, yet across the three corpora and the different reading measures, we consistently found that language-specific models developed for Dutch outperformed the multilingual models. The sole exception to this trend was observed in total reading times of the GECO corpus, for which salamandra-7b best captured participants’ processing difficulty. Our findings suggest that, in general, language-specific models have an advantage over multilingual models when it comes to psychometric predictive power. Based on this advantage, we recommend using language-specific Dutch GPT-2 models for analysing Dutch reading data: gpt2-small-dutch or gpt2-medium-dutch-embeddings (de Vries & Nissim, 2020). On the other hand, it can also be noted that the surprisal estimates of the multilingual models showed a significant effect on reading times (with some exceptions: mGPT on first fixation durations of MECO and GECO and total reading times of GECO), thereby endorsing the conclusion by Hollenstein et al. (2021) that multilingual models are capable of predicting reading times.

In addition, the largest models are not recommended for psycholinguistic modelling, as the results of the Dutch LLMs presented here corroborate the ubiquitous inverse scaling trend observed in English language models (see Fig. 2; de Varda & Marelli, 2023; Oh et al., 2022; Oh & Schuler, 2023; Steuer et al., 2023). Across corpora, the models’ surprisal estimates that result in the largest increase in model fit to reading times are from comparatively small models. However, the trend we observed in the Dutch models is less pronounced than in some previous work on English models (e.g. de Varda & Marelli, 2023; Oh & Schuler, 2023). The substantial variability in model configuration type (i.e. gpt2, gpt-neo, mistral, phi and llama configuration) and additional differences in context length, tokenizer and training procedure of the models compared here leaves open the possibility that the interplay of those factors occluded an inverse scaling trend that may well be observable within a single model family (e.g. GPT-2 small–XL; Oh et al., 2022). Fitting a linear model on the mean increase in log likelihood per model as a function of the number of parameters confirmed the inverse scaling trend. However, this effect interacted with the corpus being used for the evaluation, as the trend reversed for the GECO corpus, showing a benefit for the larger models. Recent work has revealed that the inverse scaling trend of LLMs persisted across languages on data from the MECO corpus (Oh & Schuler, 2025). However, for three out of the 12 languages, Dutch, Korean and English, this inverse scaling was not found. For Dutch, the authors resorted to mGPT to estimate surprisal. Here, we did test multiple sets of Dutch monolingual models with different sizes: gpt-neo-125-M/1.3B-dutch; gpt2-medium/large-dutch; gpt2-small/medium-dutch-finetune-oscar and gpt2-small/medium-dutch-embeddings. For all but the final set of models, the smaller variant’s surprisal estimate predicted reading times better for the MECO corpus. This further highlights the importance of a thorough investigation using language-specific models and a set of diverse corpora, as we corroborate the results of Oh and Schuler (2025) but also raise the question whether the same trend would persist when a larger context is present, a line of research for which the GECO corpora may be useful, as they allow one to test this for Chinese, Dutch and English (GECO, Cop et al., 2017; GECO-CN, Sui et al., 2023).

### Model-specific effects

A large-scale comparison like the one we present here further highlights the specificity of some models and the need to consider multiple text types and context sizes when evaluating a model’s psychometric predictive power. For the RaCCooNS and MECO corpus, the inverse scaling trend was more pronounced with the large models clustering at the low end of the performance spectrum. However, for the largest corpus used here, GECO, the largest models did noticeably better. Consider first the case of gpt2-small-dutch-finetune-oscar. This model performed best on the RaCCooNS and MECO data, while its psychometric predictive power dropped significantly on the GECO corpus. The largest model considered here (i.e. EuroLLM-9B) shows the opposite trend. Its predictive power increased across the three corpora, with the best performance on GECO. For the multilingual models, mGPT stands out in terms of variability, performing very well on RaCCooNS and MECO but terribly on GECO. This is important, as mGPT is a well-established model (Wilcox et al., 2023); using this model, for example, to investigate predictability effects in L1–L2 reading using the GECO corpus could have significantly altered the conclusions.

The question remains what exactly causes these variations in predictive power. On the one hand, it could be that the type of content the model was trained on aligned better with the text it was evaluated on. On the other hand, the larger context window of the bigger models (i.e. 8,192 tokens for Llama-3-8B-dutch vs 1,024 tokens for gpt2-small-dutch) can perhaps compensate for their declining psychometric performance, as GECO is the only corpus that allows for the full use of the context window of the models. Future endeavours investigating why some models explain reading times better need to consider the scaling of the available context when evaluating models’ predictive power. Hu et al. (2024) showed that increasing the context length resulted in a sharper probability distribution, with the largest effect on content words. Investigating the impact of gradually increasing the context length on the fit of surprisal to reading times would be an interesting direction for future work. Conducting a similar evaluation as the one presented here but systematically varying the context window of a specific model within a corpus that is large enough to allow the full use of the context window could further elucidate the conditions for which the inverse scaling trend might not persist.

Such an inquiry could offer a valuable complement to the analysis presented by Kuribayashi et al. (2022), where it was shown that the opposite operation, limiting the context length of a model, increased its psychometric predictive power. In this study, considering only the previous word for the prediction of the next yielded the best outcome. In line with these results, altering the self-attention mechanism of a transformer model to emphasize the local context resulted in a significant increase in the predictive power of GPT-2 (de Varda & Marelli, 2024). Both these results stem from cases where participants had at most a paragraph worth of context. This stands in contrast to the GECO corpus (Cop et al., 2017), presenting a narrative spanning across more than 60,000 words, potentially allowing for stronger integration of information by a reader. The length of the narrative may be an important factor driving the effect of limiting the context presented to the model. Within larger narratives (like the full novel in GECO), the positive effect of constraining the model to consider a more local context for the prediction of the next word might be diminished compared to a more ‘local’ setup of reading sentences.

Beyond the context window, the model’s tokenizer could also affect the predictive power of surprisal for a given corpus. Take for example the GECO corpus. This corpus presents reading data on a novel with above-average reading ease, containing many short and highly frequent words. For this input, the LlamaTokenizers used by the largest models (salamandra-7b, Llama-3-8B-dutch and EuroLLM-9B) split up fewer words into subword tokens compared to the MECO content (see supplementary Fig. S1). The surprisal value of subword tokens might be estimated suboptimally, causing a decline in psychometric predictive power. Teasing apart the effects of tokenization and context window size on the model’s performance is an interesting avenue for future research. Past work demonstrated that byte-pair encoding tokenization for estimating surprisal is equivalent to morphological or orthographic tokenization in terms of predictive power on reading time data (Nair & Resnik, 2023). However, it remains to be tested whether other BPE variations, departing more strongly from morphological subword tokenization, produce a comparable surprisal estimation.

### Surprisal linearity

The value of including an outlier corpus in terms of available context in a model comparison was also shown in the analysis concerning the linking function of surprisal and reading times. For both the RaCCooNS and MECO corpus, the relationship between surprisal and reading times was deemed to be linear, regardless of the model used to estimate surprisal, replicating earlier findings for English (Smith & Levy, 2013; Wilcox et al., 2023). However, in the case of GECO, a model allowing for a non-linear effect of surprisal caused a greater increase in model fit than the model forcing a linear link, but only for mGPT and gpt2-small-dutch and not when using the best-performing multilingual model (salamandra-2b). The shape of this non-linearity was visually different for each language model, with the Dutch models’ surprisal showing a distinct superlinear effect and mGPT showing mostly a sublinear effect. This results echoes the conclusion by Xu et al. (2023), who also showed a superlinear effect for Dutch, with the exact shape of the surprisal effect depending on the language model used to estimate surprisal. The source of this variation remains unclear and may warrant further investigation.

### New perspectives on the surprisal bottleneck

Recent studies have explored alternatives to surprisal when investigating the word-by-word processing difficulty experienced by readers. Brysbaert et al. (2024) showed that word familiarity estimates by an LLM accounted for lexical decision and naming performance better than traditional frequency. This raised the question of whether the effect of surprisal still stands when controlling for this LLM-based frequency measure. Relatedly, Opedal et al. (2024) argued that traditional effects of surprisal may be an overestimation of the effect of prediction during reading. Projecting surprisal on the orthogonal complement of frequency, they showed that the purely contextual predictor explains a proportion of variance far below standard surprisal. This also relates back to the previous notion of locally biased transformer models (de Varda & Marelli, 2024; Kuribayashi et al., 2022). In its most extreme form, a locally biased transformer simply estimates unigram probability which is proportional to frequency. Future studies could investigate the interplay between these predictors, which combination yields the greatest increase in predictive power for reading times, and what novel measures like LLM-estimated familiarity mean for the processing difficulty experienced by readers.

## Conclusion

To conclude, this paper presents a comprehensive evaluation of a variety of Dutch large language models in reference to well-established multilingual models. As the first resource of its kind, it offers a valuable tool for researchers looking for a psychometrically valid language model of Dutch. Furthermore, the results presented here underscore that LLM-based surprisal estimates require careful scrutiny, as the fit to reading times was highly model- and data-specific. In addition, researchers are invited to make use of the surprisal estimates and the method to extract those, both of which are documented on OSF (https://osf.io/wr4qf/).

## Supplementary Information

Below is the link to the electronic supplementary material.

Supplementary file1 (PDF 393 kb)

## Data Availability

Materials and data are available at https://osf.io/wr4qf/. None of the reported studies were preregistered.

## Footnotes

1 https://huggingface.co/ is a platform for building and sharing machine learning applications and datasets.

2 Note that the corresponding Hugging Face space https://huggingface.co/spaces/BramVanroy/open_dutch_llm_leaderboard is no longer maintained. Users are referred to the ScandEval benchmarks for Dutch instead (https://scandeval.com/dutch-nlg/).

3 Baseline model: lm(ET measure ~ 1 + Freq × Word_length + prev_Freq × prev_Word_length + Word_position). Full model: lm(ET measure ~ 1 + Freq × Word_length + prev_Freq × prev_Word_length + Word_position + *Surprisal* + *prev_Surprisal*).

4 Baseline: gaze duration ~ te(frequency, word_length, bs = ‘cr’) + te(prev_frequency, prev_word_length, bs = ‘cr’). Linear: gaze duration ~ surprisal + prev_surprisal + te(frequency, word_length, bs = ‘cr’) + te(prev_frequency, prev_word_length, bs = ‘cr’). Non-linear: gaze duration ~ s(surprisal, bs = ‘cr’, k = 6) + s(prev_surprisal, bs = ‘cr’, k = 6) + te(frequency, word_length, bs = ‘cr’) + te(prev_frequency, prev_word_length, bs = ‘cr’).

5 lm(loglikelihood increase ~ corpus + ET measure + parameters + parameters:corpus + parameters:model_family).

## Code availability

Code to extract surprisal estimates and analysis code are available on OSF: https://osf.io/wr4qf/.
