# Supplementary material for: A systematic evaluation of Dutch large language models’ surprisal estimates in sentence, paragraph and book reading
Source: Behav Res Methods. 2025 Aug 18;57(9):266. doi: 10.3758/s13428-025-02774-4 (PMC12361287; doi:10.3758/s13428-025-02774-4)
Supplement: Supplementary file 1 — Supplementary file1 (PDF 393 kb) [file 13428_2025_2774_MOESM1_ESM.pdf]

Supplementary Material

Table S1 Effect of Tokenizer

	Tokenizer type	RaCCooNS		MECO		GECO	
		Prop. subword tokenization	# tokens	Prop. subword tokenization	# tokens	Prop. subword tokenization	# tokens
**EuroLLM-9B**	LlamaTokenizer	.12	2666	.22	2554	.17	58.401
**Llama-3-8B-dutch**	LlamaTokenizer	.32	3374	.41	3324	.33	71.697
**salamandra-7b**	LlamaTokenizer	.09	2584	.20	2513	.14	56.259
**tweety-7b-dutch-v24a**	GPT2Tokenizer	.003	2322	.096	2180	.029	48.947
**Boreas-7B**	LlamaTokenizer	.43	3785	.46	3608	.44	80.632
**GEITje-7B**	LlamaTokenizer	.43	3785	.46	3608	.44	80.632
**fietje-2**	CodeGenTokenizer	.61	4382	.63	4202	.61	91.857
**salamandra-2b**	LlamaTokenizer	.09	2584	.20	2513	.14	58.401
**EuroLLM-1.7B**	LlamaTokenizer	.12	2666	.22	2554	.17	56.259
**mGPT**	GPT2Tokenizer	.22	2979	.28	2759	.25	63.850
**gpt-neo-1.3B-dutch**	GPT2Tokenizer	.0030	2322	.096	2180	.029	48.947
**gpt2-large-dutch**	GPT2Tokenizer	.0030	2322	.096	2180	.029	48.947
**gpt2-medium-dutch**	GPT2Tokenizer	.0030	2322	.096	2180	.029	48.947
**gpt2-medium-dutch-finetune-oscar**	GPT2Tokenizer	.013	2345	.096	2200	.071	51.829
**gpt2-medium-dutch-embeddings**	GPT2Tokenizer	.0052	2328	.10	2189	.044	49.840
**gpt-neo-125M-dutch**	GPT2Tokenizer	.0030	2322	.096	2180	.029	48.947
**gpt2-small-dutch-finetune-oscar**	GPT2Tokenizer	.013	2345	.096	2200	.071	51.829
**gpt2-small-dutch**	GPT2Tokenizer	.0052	2328	.10	2189	.044	49.840
**gpt2-small-dutch-embeddings**	GPT2Tokenizer	.0052	2328	.10	2189	.044	49.840

Note: **Prop. subword tokenization** denotes the proportion of words (i.e., areas of interest) in the text stimuli for which the tokenization did not align with the word boundary. In other words, the proportion of words processed as more than one token. **# tokens** shows the total number of tokens used to process the corpus text. Prior to calculating these summary statistics, the sentence initial words and words attached to punctuation were removed, following the main analyses. This resulted in 2315 remaining words for RaCCooNS, 1907 for MECO and 46.560 for GECO.

For Review Only

Table S2 Estimates of Psychometric Predictive Power

Model	Corpus	Eye-Tracking Measure	ΔlogLik per 1000 words	95% Confidence Interval	p-value
**5-Gram**	RaCCooNS	First Fixation Duration	1.32	[1.03; 1.61]	***
**gpt2-small-dutch-embeddings**	RaCCooNS	First Fixation Duration	0.85	[0.42; 1.28]	***
**gpt2-small-dutch**	RaCCooNS	First Fixation Duration	0.75	[0.35; 1.16]	***
**gpt2-small-dutch-finetune-oscar**	RaCCooNS	First Fixation Duration	0.81	[0.38; 1.24]	***
**gpt-neo-125M-dutch**	RaCCooNS	First Fixation Duration	0.77	[0.37; 1.17]	***
**gpt2-medium-dutch-embeddings**	RaCCooNS	First Fixation Duration	0.65	[0.31; 1]	***
**gpt2-medium-dutch-finetune-oscar**	RaCCooNS	First Fixation Duration	0.76	[0.42; 1.09]	***
**gpt2-medium-dutch**	RaCCooNS	First Fixation Duration	0.60	[0.24; 0.96]	***
**gpt2-large-dutch**	RaCCooNS	First Fixation Duration	0.64	[0.26; 1.03]	***
**gpt-neo-1.3B-dutch**	RaCCooNS	First Fixation Duration	0.47	[0.1; 0.85]	***
**mGPT**	RaCCooNS	First Fixation Duration	0.42	[0.13; 0.71]	***
**EuroLLM-1.7B**	RaCCooNS	First Fixation Duration	0.41	[0.1; 0.71]	**
**salamandra-2b**	RaCCooNS	First Fixation Duration	0.42	[0.15; 0.7]	**
**fietje-2**	RaCCooNS	First Fixation Duration	0.48	[0.14; 0.82]	***
**GEITje-7B**	RaCCooNS	First Fixation Duration	0.32	[0.03; 0.6]	*
**Boreas-7B**	RaCCooNS	First Fixation Duration	0.41	[0.09; 0.72]	**
**tweety-7b-dutch-v24a**	RaCCooNS	First Fixation Duration	0.08	[-0.08; 0.24]	NS.
**salamandra-7b**	RaCCooNS	First Fixation Duration	0.29	[-0.02; 0.6]	*
**Llama-3-8B-dutch**	RaCCooNS	First Fixation Duration	0.41	[0.11; 0.71]	**
**EuroLLM-9B**	RaCCooNS	First Fixation Duration	0.39	[0.06; 0.71]	**
**5-Gram**	MECO	First Fixation Duration	0.22	[0.08; 0.36]	NS.
**gpt2-small-dutch-embeddings**	MECO	First Fixation Duration	0.43	[0.11; 0.75]	*
**gpt2-small-dutch**	MECO	First Fixation Duration	0.43	[0.09; 0.78]	*
**gpt2-small-dutch-finetune-oscar**	MECO	First Fixation Duration	0.34	[-0.05; 0.73]	*
**gpt-neo-125M-dutch**	MECO	First Fixation Duration	0.52	[-0.01; 1.05]	**
**gpt2-medium-dutch-embeddings**	MECO	First Fixation Duration	0.40	[0.11; 0.68]	*
**gpt2-medium-dutch-finetune-oscar**	MECO	First Fixation Duration	0.32	[0.01; 0.63]	NS.
**gpt2-medium-dutch**	MECO	First Fixation Duration	0.57	[0.01; 1.13]	***
**gpt2-large-dutch**	MECO	First Fixation Duration	0.35	[-0.12; 0.83]	*

For Review Only

Table S2 Estimates of Psychometric Predictive Power

Model	Corpus	Eye-Tracking Measure	ΔlogLik per 1000 words	95% Confidence Interval	p-value
**gpt-neo-1.3B-dutch**	MECO	First Fixation Duration	0.39	[-0.06; 0.84]	*
**mGPT**	MECO	First Fixation Duration	0.34	[-0.08; 0.75]	*
**EuroLLM-1.7B**	MECO	First Fixation Duration	0.23	[-0.14; 0.61]	NS.
**salamandra-2b**	MECO	First Fixation Duration	0.22	[-0.14; 0.59]	NS.
**fietje-2**	MECO	First Fixation Duration	0.12	[-0.24; 0.48]	NS.
**GEITje-7B**	MECO	First Fixation Duration	0.11	[-0.15; 0.36]	NS.
**Boreas-7B**	MECO	First Fixation Duration	0.21	[-0.1; 0.51]	NS.
**tweety-7b-dutch-v24a**	MECO	First Fixation Duration	0.20	[-0.11; 0.51]	NS.
**salamandra-7b**	MECO	First Fixation Duration	0.16	[-0.1; 0.43]	NS.
**Llama-3-8B-dutch**	MECO	First Fixation Duration	0.13	[-0.1; 0.36]	NS.
**EuroLLM-9B**	MECO	First Fixation Duration	0.13	[-0.13; 0.39]	NS.
**5-Gram**	GECO	First Fixation Duration	0.60	[0.43; 0.76]	***
**gpt2-small-dutch-embeddings**	GECO	First Fixation Duration	0.82	[0.61; 1.03]	***
**gpt2-small-dutch**	GECO	First Fixation Duration	0.61	[0.36; 0.86]	***
**gpt2-small-dutch-finetune-oscar**	GECO	First Fixation Duration	0.31	[0.24; 0.38]	***
**gpt-neo-125M-dutch**	GECO	First Fixation Duration	0.17	[0.08; 0.27]	***
**gpt2-medium-dutch-embeddings**	GECO	First Fixation Duration	0.84	[0.66; 1.02]	***
**gpt2-medium-dutch-finetune-oscar**	GECO	First Fixation Duration	0.45	[0.34; 0.55]	***
**gpt2-medium-dutch**	GECO	First Fixation Duration	0.28	[0.19; 0.37]	***
**gpt2-large-dutch**	GECO	First Fixation Duration	0.31	[0.19; 0.43]	***
**gpt-neo-1.3B-dutch**	GECO	First Fixation Duration	0.06	[0.01; 0.11]	NS.
**mGPT**	GECO	First Fixation Duration	0.05	[-0.01; 0.11]	NS.
**EuroLLM-1.7B**	GECO	First Fixation Duration	0.75	[0.49; 1.01]	***
**salamandra-2b**	GECO	First Fixation Duration	0.79	[0.54; 1.04]	***
**fietje-2**	GECO	First Fixation Duration	0.73	[0.49; 0.97]	***
**GEITje-7B**	GECO	First Fixation Duration	0.53	[0.35; 0.72]	***
**Boreas-7B**	GECO	First Fixation Duration	0.68	[0.45; 0.91]	***
**tweety-7b-dutch-v24a**	GECO	First Fixation Duration	0.68	[0.46; 0.9]	***
**salamandra-7b**	GECO	First Fixation Duration	0.72	[0.46; 0.97]	***

For Review Only

Table S2 Estimates of Psychometric Predictive Power

Model	Corpus	Eye-Tracking Measure	ΔlogLik per 1000 words	95% Confidence Interval	p-value
**Llama-3-8B-dutch**	GECO	First Fixation Duration	0.61	[0.39; 0.83]	***
**EuroLLM-9B**	GECO	First Fixation Duration	0.69	[0.47; 0.92]	***
**5-Gram**	RaCCooNS	Gaze Duration	1.38	[0.85; 1.9]	***
**gpt2-small-dutch-embeddings**	RaCCooNS	Gaze Duration	1.16	[0.44; 1.88]	***
**gpt2-small-dutch**	RaCCooNS	Gaze Duration	1.13	[0.42; 1.83]	***
**gpt2-small-dutch-finetune-oscar**	RaCCooNS	Gaze Duration	1.40	[0.76; 2.03]	***
**gpt-neo-125M-dutch**	RaCCooNS	Gaze Duration	1.30	[0.69; 1.92]	***
**gpt2-medium-dutch-embeddings**	RaCCooNS	Gaze Duration	1.01	[0.42; 1.6]	***
**gpt2-medium-dutch-finetune-oscar**	RaCCooNS	Gaze Duration	1.21	[0.68; 1.74]	***
**gpt2-medium-dutch**	RaCCooNS	Gaze Duration	1.03	[0.49; 1.57]	***
**gpt2-large-dutch**	RaCCooNS	Gaze Duration	1.11	[0.54; 1.68]	***
**gpt-neo-1.3B-dutch**	RaCCooNS	Gaze Duration	0.79	[0.21; 1.37]	***
**mGPT**	RaCCooNS	Gaze Duration	0.99	[0.53; 1.45]	***
**EuroLLM-1.7B**	RaCCooNS	Gaze Duration	0.82	[0.35; 1.3]	***
**salamandra-2b**	RaCCooNS	Gaze Duration	1.02	[0.59; 1.44]	***
**fietje-2**	RaCCooNS	Gaze Duration	0.70	[0.21; 1.19]	***
**GEITje-7B**	RaCCooNS	Gaze Duration	0.51	[0.07; 0.95]	***
**Boreas-7B**	RaCCooNS	Gaze Duration	0.64	[0.29; 1]	***
**tweety-7b-dutch-v24a**	RaCCooNS	Gaze Duration	0.08	[-0.09; 0.24]	NS.
**salamandra-7b**	RaCCooNS	Gaze Duration	0.68	[0.17; 1.19]	***
**Llama-3-8B-dutch**	RaCCooNS	Gaze Duration	0.69	[0.22; 1.16]	***
**EuroLLM-9B**	RaCCooNS	Gaze Duration	0.72	[0.26; 1.19]	***
**5-Gram**	MECO	Gaze Duration	0.51	[-0.11; 1.12]	***
**gpt2-small-dutch-embeddings**	MECO	Gaze Duration	2.03	[1.43; 2.64]	***
**gpt2-small-dutch**	MECO	Gaze Duration	2.25	[1.58; 2.93]	***
**gpt2-small-dutch-finetune-oscar**	MECO	Gaze Duration	2.57	[1.62; 3.52]	***
**gpt-neo-125M-dutch**	MECO	Gaze Duration	2.28	[1.31; 3.24]	***
**gpt2-medium-dutch-embeddings**	MECO	Gaze Duration	2.14	[1.3; 2.98]	***
**gpt2-medium-dutch-finetune-oscar**	MECO	Gaze Duration	2.42	[1.26; 3.58]	***

For Review Only

Table S2 Estimates of Psychometric Predictive Power

Model	Corpus	Eye-Tracking Measure	ΔlogLik per 1000 words	95% Confidence Interval	p-value
**gpt2-medium-dutch**	MECO	Gaze Duration	2.22	[1.33; 3.12]	***
**gpt2-large-dutch**	MECO	Gaze Duration	1.98	[1.07; 2.89]	***
**gpt-neo-1.3B-dutch**	MECO	Gaze Duration	1.73	[0.95; 2.51]	***
**mGPT**	MECO	Gaze Duration	2.35	[1.34; 3.36]	***
**EuroLLM-1.7B**	MECO	Gaze Duration	1.34	[0.6; 2.08]	***
**salamandra-2b**	MECO	Gaze Duration	1.51	[0.78; 2.24]	***
**fietje-2**	MECO	Gaze Duration	1.26	[0.47; 2.05]	***
**GEITje-7B**	MECO	Gaze Duration	0.80	[0.13; 1.47]	***
**Boreas-7B**	MECO	Gaze Duration	1.12	[0.53; 1.72]	***
**tweety-7b-dutch-v24a**	MECO	Gaze Duration	2.09	[1.48; 2.7]	***
**salamandra-7b**	MECO	Gaze Duration	1.16	[0.45; 1.87]	***
**Llama-3-8B-dutch**	MECO	Gaze Duration	1.22	[0.42; 2.03]	***
**EuroLLM-9B**	MECO	Gaze Duration	1.09	[0.39; 1.8]	***
**5-Gram**	GECO	Gaze Duration	0.94	[0.79; 1.1]	***
**gpt2-small-dutch-embeddings**	GECO	Gaze Duration	2.33	[1.64; 3.03]	***
**gpt2-small-dutch**	GECO	Gaze Duration	1.81	[1.1; 2.52]	***
**gpt2-small-dutch-finetune-oscar**	GECO	Gaze Duration	0.78	[0.57; 1]	***
**gpt-neo-125M-dutch**	GECO	Gaze Duration	0.98	[0.7; 1.26]	***
**gpt2-medium-dutch-embeddings**	GECO	Gaze Duration	2.47	[1.82; 3.12]	***
**gpt2-medium-dutch-finetune-oscar**	GECO	Gaze Duration	0.97	[0.72; 1.22]	***
**gpt2-medium-dutch**	GECO	Gaze Duration	1.11	[0.74; 1.47]	***
**gpt2-large-dutch**	GECO	Gaze Duration	1.19	[0.83; 1.54]	***
**gpt-neo-1.3B-dutch**	GECO	Gaze Duration	0.45	[0.29; 0.61]	***
**mGPT**	GECO	Gaze Duration	0.08	[-0.01; 0.17]	***
**EuroLLM-1.7B**	GECO	Gaze Duration	1.69	[1.12; 2.27]	***
**salamandra-2b**	GECO	Gaze Duration	1.72	[1.22; 2.22]	***
**fietje-2**	GECO	Gaze Duration	1.69	[1.15; 2.23]	***
**GEITje-7B**	GECO	Gaze Duration	1.19	[0.82; 1.56]	***
**Boreas-7B**	GECO	Gaze Duration	1.47	[0.96; 1.99]	***

For Review Only

Table S2 Estimates of Psychometric Predictive Power

Model	Corpus	Eye-Tracking Measure	ΔlogLik per 1000 words	95% Confidence Interval	p-value
**tweety-7b-dutch-v24a**	GECO	Gaze Duration	1.41	[0.98; 1.85]	***
**salamandra-7b**	GECO	Gaze Duration	1.59	[1.06; 2.12]	***
**Llama-3-8B-dutch**	GECO	Gaze Duration	1.47	[1; 1.95]	***
**EuroLLM-9B**	GECO	Gaze Duration	1.53	[1; 2.07]	***
**5-Gram**	RaCCooNS	Total Reading Time	2.25	[1.88; 2.63]	***
**gpt2-small-dutch-embeddings**	RaCCooNS	Total Reading Time	2.96	[2.5; 3.43]	***
**gpt2-small-dutch**	RaCCooNS	Total Reading Time	2.92	[2.51; 3.34]	***
**gpt2-small-dutch-finetune-oscar**	RaCCooNS	Total Reading Time	2.68	[2.24; 3.12]	***
**gpt-neo-125M-dutch**	RaCCooNS	Total Reading Time	2.79	[2.42; 3.16]	***
**gpt2-medium-dutch-embeddings**	RaCCooNS	Total Reading Time	2.74	[2.32; 3.16]	***
**gpt2-medium-dutch-finetune-oscar**	RaCCooNS	Total Reading Time	2.94	[2.53; 3.35]	***
**gpt2-medium-dutch**	RaCCooNS	Total Reading Time	3.29	[2.84; 3.75]	***
**gpt2-large-dutch**	RaCCooNS	Total Reading Time	3.38	[2.94; 3.81]	***
**gpt-neo-1.3B-dutch**	RaCCooNS	Total Reading Time	2.66	[2.28; 3.03]	***
**mGPT**	RaCCooNS	Total Reading Time	2.37	[1.78; 2.97]	***
**EuroLLM-1.7B**	RaCCooNS	Total Reading Time	2.57	[2.21; 2.93]	***
**salamandra-2b**	RaCCooNS	Total Reading Time	2.88	[2.4; 3.36]	***
**fietje-2**	RaCCooNS	Total Reading Time	3.00	[2.44; 3.57]	***
**GEITje-7B**	RaCCooNS	Total Reading Time	3.09	[2.34; 3.84]	***
**Boreas-7B**	RaCCooNS	Total Reading Time	3.22	[2.43; 4]	***
**tweety-7b-dutch-v24a**	RaCCooNS	Total Reading Time	0.19	[0; 0.37]	NS.
**salamandra-7b**	RaCCooNS	Total Reading Time	2.80	[2.18; 3.42]	***
**Llama-3-8B-dutch**	RaCCooNS	Total Reading Time	3.03	[2.29; 3.76]	***
**EuroLLM-9B**	RaCCooNS	Total Reading Time	2.82	[2.33; 3.32]	***
**5-Gram**	MECO	Total Reading Time	0.56	[0.26; 0.86]	**
**gpt2-small-dutch-embeddings**	MECO	Total Reading Time	3.73	[2.86; 4.61]	***
**gpt2-small-dutch**	MECO	Total Reading Time	4.31	[3.16; 5.47]	***
**gpt2-small-dutch-finetune-oscar**	MECO	Total Reading Time	4.91	[3.41; 6.41]	***
**gpt-neo-125M-dutch**	MECO	Total Reading Time	4.98	[3.8; 6.15]	***

For Review Only

Table S2 Estimates of Psychometric Predictive Power

Model	Corpus	Eye-Tracking Measure	ΔlogLik per 1000 words	95% Confidence Interval	p-value
**gpt2-medium-dutch-embeddings**	MECO	Total Reading Time	3.84	[2.88; 4.8]	***
**gpt2-medium-dutch-finetune-oscar**	MECO	Total Reading Time	5.27	[3.92; 6.61]	***
**gpt2-medium-dutch**	MECO	Total Reading Time	4.78	[3.85; 5.72]	***
**gpt2-large-dutch**	MECO	Total Reading Time	4.69	[3.75; 5.64]	***
**gpt-neo-1.3B-dutch**	MECO	Total Reading Time	4.79	[3.88; 5.7]	***
**mGPT**	MECO	Total Reading Time	4.25	[3; 5.51]	***
**EuroLLM-1.7B**	MECO	Total Reading Time	2.22	[1.41; 3.03]	***
**salamandra-2b**	MECO	Total Reading Time	2.93	[1.95; 3.9]	***
**fietje-2**	MECO	Total Reading Time	2.89	[1.95; 3.83]	***
**GEITje-7B**	MECO	Total Reading Time	2.75	[2.08; 3.42]	***
**Boreas-7B**	MECO	Total Reading Time	2.53	[1.68; 3.39]	***
**tweety-7b-dutch-v24a**	MECO	Total Reading Time	4.60	[2.97; 6.23]	***
**salamandra-7b**	MECO	Total Reading Time	2.68	[1.98; 3.38]	***
**Llama-3-8B-dutch**	MECO	Total Reading Time	2.75	[2.01; 3.49]	***
**EuroLLM-9B**	MECO	Total Reading Time	2.11	[1.48; 2.73]	***
**5-Gram**	GECO	Total Reading Time	2.26	[2.07; 2.45]	***
**gpt2-small-dutch-embeddings**	GECO	Total Reading Time	5.13	[4.24; 6.02]	***
**gpt2-small-dutch**	GECO	Total Reading Time	4.33	[3.32; 5.34]	***
**gpt2-small-dutch-finetune-oscar**	GECO	Total Reading Time	2.10	[1.67; 2.52]	***
**gpt-neo-125M-dutch**	GECO	Total Reading Time	1.34	[0.92; 1.76]	***
**gpt2-medium-dutch-embeddings**	GECO	Total Reading Time	5.24	[4.38; 6.09]	***
**gpt2-medium-dutch-finetune-oscar**	GECO	Total Reading Time	2.02	[1.55; 2.48]	***
**gpt2-medium-dutch**	GECO	Total Reading Time	1.81	[1.28; 2.33]	***
**gpt2-large-dutch**	GECO	Total Reading Time	2.12	[1.57; 2.66]	***
**gpt-neo-1.3B-dutch**	GECO	Total Reading Time	0.41	[0.19; 0.63]	***
**mGPT**	GECO	Total Reading Time	0.01	[-0.02; 0.04]	NS.
**EuroLLM-1.7B**	GECO	Total Reading Time	5.01	[4.46; 5.56]	***
**salamandra-2b**	GECO	Total Reading Time	5.12	[4.75; 5.49]	***
**fietje-2**	GECO	Total Reading Time	5.26	[4.79; 5.73]	***

For Review Only

Table S2 Estimates of Psychometric Predictive Power

Model	Corpus	Eye-Tracking Measure	ΔlogLik per 1000 words	95% Confidence Interval	p-value
**GEITje-7B**	GECO	Total Reading Time	4.62	[4.23; 5.01]	***
**Boreas-7B**	GECO	Total Reading Time	5.27	[4.68; 5.85]	***
**tweety-7b-dutch-v24a**	GECO	Total Reading Time	4.57	[3.93; 5.21]	***
**salamandra-7b**	GECO	Total Reading Time	5.44	[4.86; 6.03]	***
**Llama-3-8B-dutch**	GECO	Total Reading Time	4.77	[4.32; 5.21]	***
**EuroLLM-9B**	GECO	Total Reading Time	5.16	[4.62; 5.7]	***

Note: Mean increase in log likelihood over the five folds of the cross validation. P-values were calculated using Fisher's combined probability test. p-value < .001: ***; < .01: **; < .05: *; >.05: NS.

For Review Only

Figure S1 Illustration of Different Tokenization Algorithms

MECO

A.

Janus is in de oude Romeinse religie en mythes de god van het begin
en van poorten. Hij heeft een dubbele aard en wordt meestal
voorgesteld met twee gezichten, omdat hij zowel naar de toekomst als
naar het verleden kijkt. Janus overzag het begin en het einde van
conflicten, en vandaar oorlog en vrede. De deuren van zijn tempel
waren open in tijden van oorlog en gesloten in tijden van vrede. Als
god van de poorten werd hij ook geassocieërd met het betreden en
verlaten van huisdeuren.

B.

Janus is in de oude Romeinse religie en mythes de god van het begin en
van poorten. Hij heeft een dubbele aard en wordt meestal voorgesteld
met twee gezichten, omdat hij zowel naar de toekomst als naar het ver
leden kijkt. Janus overzag het begin en het einde van conflicten, en v
andaar oorlog en vrede. De deuren van zijn tempel waren open in tijden
van oorlog en gesloten in tijden van vrede. Als god van de poorten
werd hij ook geassocieërd met het betreden en verlaten van huisdeuren
.

GECO

C.

De enorme belangstelling die het publiek toonde voor wat indertijd
bekend stond als 'de zaak Styles', wordt nu wat minder. Niettemin
is mij, zowel door mijn vriend Poirot als door de betrokken
familieleden, verzocht een verslag van het hele gebeuren te
schrijven, gezien het feit dat er in de hele wereld grote rucht
baarheid aan gegeven is. Op deze manier hopen we voorgoed een einde
te maken aan de sensationele geruchten die nog steeds de ronde doen
. Ik zal daarom in het kort de omstandigheden uiteenzetten die ertoe
geleid hebben dat ik bij de zaak betrokken raakte.

D.

De enorme belangstelling die het publiek toonde voor wat indertijd
bekend stond als 'de zaak Styles', wordt nu wat minder. Niettemin
is mij, zowel door mijn vriend Poirot als door de betrokken familie
leden, verzocht een verslag van het hele gebeuren te schrijven, gez
ien het feit dat er in de hele wereld grote ruchtbaarheid aan gege
ven is. Op deze manier hopen we voorgoed een einde te maken aan de
sensationele geruchten die nog steeds de ronde doen. Ik zal daarom
in het kort de omstandigheden uiteenzetten die ertoe geleid hebben
dat ik bij de zaak betrokken raakte.

Note: Visual representation of different tokenizers processing textual input. Each colour chunk represents a separate token. From the MECO corpus, 87 words from the 'Janus' text were used as an example. **A.** gpt2-medium-dutch-embeddings tokenizer (GPT2Tokenizer): 102 tokens were used to process this text expert. **B.** Llama-3-8B-dutch (LlamaTokenizer): 151 tokens were necessary to represent the same text. For GECO, the first 96 words of the corpus are used as an example. **C.** gpt2-medium-dutch-embeddings tokenizer (GPT2Tokenizer). A total of 111 tokens is used for the text of 96 words. **D**. Llama-3-8B-dutch (LlamaTokenizer). A total of 164 tokens was used to represent the same input. Illustration made using Tokenizer Playground (*https://huggingface.co/spaces/Xenova/the-tokenizer-playground*)
